# Healthcare professionals' perception of the ketogenic diet among patients with chronic obstructive pulmonary disease: a cross-sectional study

**DOI:** 10.3389/fnut.2025.1558151

**Published:** 2025-06-03

**Authors:** Saleha Alqarni, Eatedal Eenizan Alsaeedi, Rayan A. Siraj, Yousef Saad Aldabayan, Amal Ismael Abdelhafez

**Affiliations:** ^1^Department of Clinical Nutrition, College of Applied Medical Sciences, King Faisal University, Al Ahsa, Saudi Arabia; ^2^Department of Clinical Nutrition, College of Applied Medical Sciences, University of Hafr Al Batin, Hafr Al Batin, Saudi Arabia; ^3^Department of Respiratory Therapy, College of Applied Medical Sciences, King Faisal University, Al Ahsa, Saudi Arabia; ^4^Critical Care and Emergency Nursing Department, Faculty of Nursing, Assiut University, Assiut, Egypt; ^5^Department of Nursing, College of Applied Medical Sciences, King Faisal University, Al Ahsa, Saudi Arabia

**Keywords:** pulmonary disease, inflammation, ketogenic diet, healthcare professionals, nutrition

## Abstract

**Background:**

Chronic obstructive pulmonary disease (COPD) is a progressive respiratory disorder characterized by persistent inflammation and airflow limitation. The ketogenic diet (KD), recognized for its anti-inflammatory properties, has potential therapeutic benefits for COPD management. However, healthcare professionals' perceptions of KD's efficacy and applicability in COPD care remain underexplored, particularly in Saudi Arabia.

**Methods:**

A cross-sectional online survey was conducted between June and September 2024, targeting healthcare professionals involved in COPD management. The survey evaluated perceptions of KD's benefits, limitations, and current nutritional practices. Descriptive statistics and logistic regression analyses were performed using JASP to identify predictors of KD training uptake and the likelihood of discussing dietary interventions with COPD patients.

**Results:**

A total of 1,068 healthcare professionals participated in the survey. Of these, 58% believed KD could improve quality of life in COPD patients, and 61% acknowledged its potential to reduce inflammation. Logistic regression identified familiarity with KD as significant predictor for receiving KD training (*p* < 0.001). Concerns regarding KD's adverse effects, such as constipation and dehydration, were noted by 76% of respondents. Only 14% reported recommending KD, citing insufficient evidence and lack of professional training as primary barriers. Additionally, 74% highlighted patient adherence challenges due to KD's restrictive nature and potential side effects.

**Conclusion:**

KD shows promise as a complementary therapy for COPD by modulating inflammation and improving symptom management. Addressing barriers such as limited evidence and inadequate professional training is essential. Further research is required to establish the efficacy and safety of KD in COPD care.

## 1 Introduction

Chronic obstructive pulmonary disease (COPD) is a progressive and debilitating respiratory condition that ranks as the fourth leading cause of death globally, affecting over 300 million individuals worldwide (1, 2). The progression of COPD is largely driven by persistent inflammation, marked by increased immune cell activity (e.g., macrophages, neutrophils, and lymphocytes) and overproduction of pro-inflammatory cytokines (e.g., TNF-α, IL-1β, IL-6), which contribute to resistance to standard therapies, such as corticosteroids (3, 4). These inflammatory processes also drive chronic airflow limitations caused by lung tissue destruction and airway obstruction, resulting in severe symptoms such as dyspnea, fatigue, and reduced physical capacity (5).

**Figure 1:**
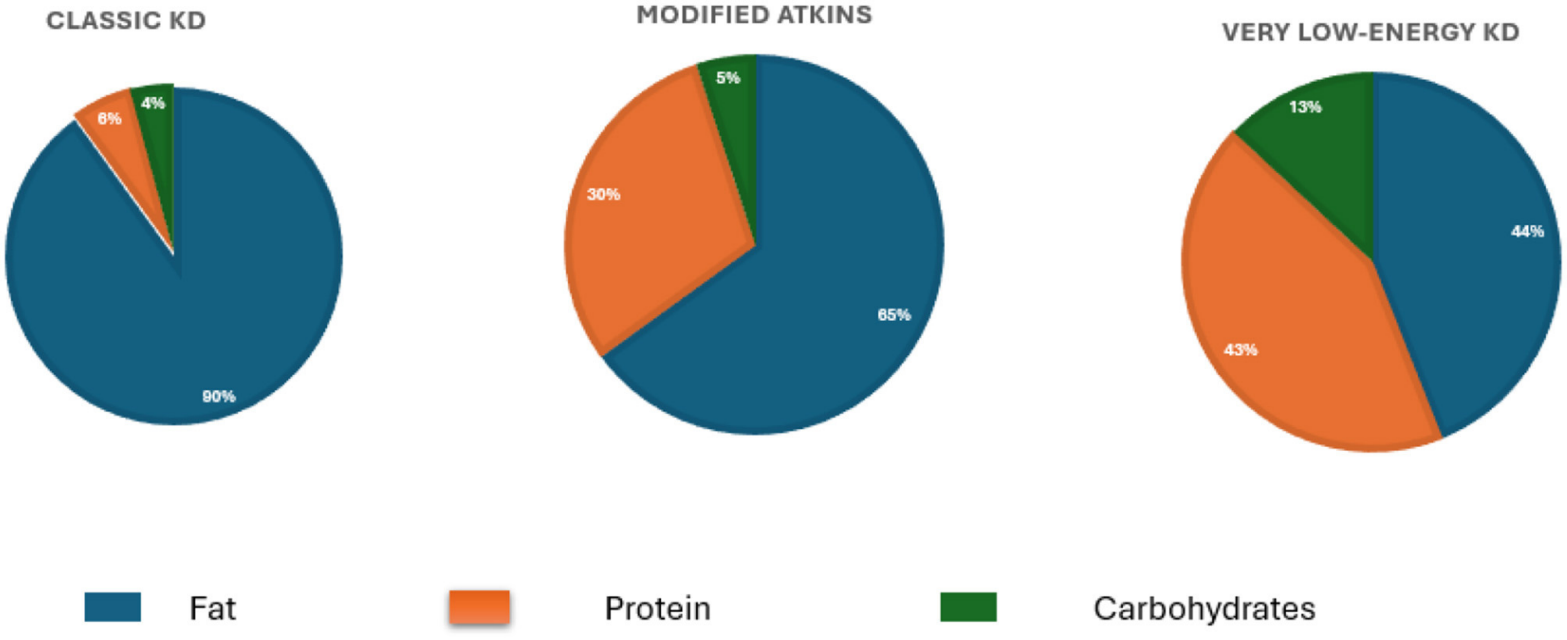
Macronutrient distribution across three types of ketogenic diets. The Classic Ketogenic Diet (KD) consists of ~90% fat, 6% protein, and 4% carbohydrates and is traditionally used in epilepsy management. The Modified Atkins Diet, introduced as a more flexible approach, includes 65% fat, 30% protein, and 5% carbohydrates. The Very Low-Energy Ketogenic Diet (VLEKD) mimics the metabolic effects of fasting and consists of 44% fat, 43% protein, and 13% carbohydrates, with a total daily energy intake typically below 800 kcal.

Despite significant advancements in COPD management, existing therapeutic strategies primarily target symptom relief, with limited focus on addressing the underlying inflammatory mechanisms. Resistance to corticosteroids, along with the limited success of other targeted therapies, underscores the urgent need for innovative approaches that offer broader anti-inflammatory effects (6, 7). One such promising approach is the ketogenic diet (KD), a high-fat, low-carbohydrate dietary regimen with known anti-inflammatory properties (8, 9). The various formulation of KD and their macronutrient compositions are illustrated in Figure 1. The KD, which has been used for nearly a century to manage and reduce seizure frequency in pediatric epilepsy (10), is now being explored for its therapeutic potential in a range of chronic conditions. Research has demonstrated that KD can reduce inflammatory markers and improve metabolic efficiency in diseases such as cardiovascular risk factors (11), Parkinson's disease (12), type II diabetes (13, 14), polycystic ovarian syndrome (15), metabolic syndrome (16), lipedema (17, 18), Alzheimer's disease (19), and several mental health disorders (20). Although the KD has demonstrated therapeutic benefits in various conditions, it is not without potential side effects, particularly during the initial adaptation phase. Common short-term symptoms include “keto flu,” which may include headaches, fatigue, nausea, dizziness, and irritability due to sudden changes in electrolyte and glucose balance (21). Gastrointestinal disturbances such as constipation and bloating are also common, potentially related to low fiber intake (22). In some cases, prolonged adherence to the KD may lead to increased low-density lipoprotein cholesterol (23), and an increased risk of kidney stones (24). Therefore, medical supervision and individualized planning are highly recommended when starting or continuing a KD.

Emerging evidence suggests that KD may benefit COPD management by targeting inflammation. For instance, a recent case report documented a 37.5% improvement in forced expiratory volume (FEV1) and normalization of inflammatory markers in a COPD patient following a KD (25). Additionally, a controlled trial involving 60 COPD patients found significant improvements in lung function with a low-carbohydrate diet, highlighting the potential of dietary interventions in managing COPD (26).

**Figure 2:**
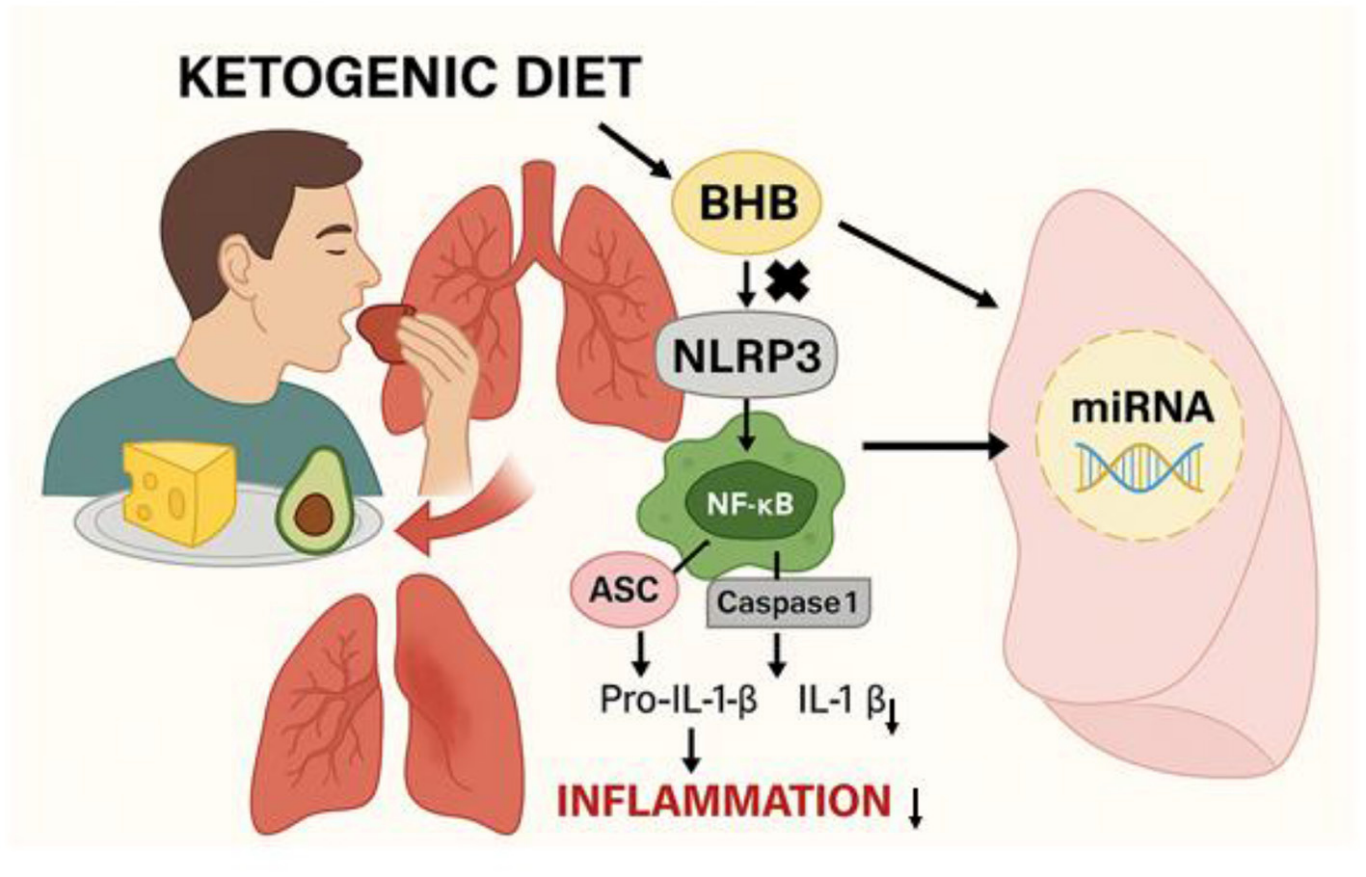
Illustration of the proposed mechanism by which the ketogenic diet modulates inflammation in COPD. A high-fat, low-carbohydrate intake induces β-hydroxybutyrate (BHB) production, which inhibits the NLRP3 inflammasome pathway. This results in reduced activation of NF-κB and decreased IL-1β production, ultimately lowering inflammation. Additionally, BHB may influence the expression of specific microRNAs (miRNAs) involved in immune regulation, contributing to improved lung function.

Recent studies have also begun to unravel the biological mechanisms underlying these benefits, particularly the role of β-hydroxybutyrate (BHB), a ketone body produced during KD. BHB inhibits the NLRP3 inflammasome, a key component in the inflammatory response associated with COPD exacerbations (27, 28). Elevated levels of NLRP3 and associated markers (Asc, caspase-1 mRNAs) have been observed in active COPD cases, making this pathway a promising target for therapeutic intervention (29). By inhibiting NLRP3, BHB may reduce systemic inflammation, as seen in other inflammatory conditions like gout (30). Emerging evidence also indicates that specific microRNAs are involved in the regulation of inflammation during KD, potentially contributing to the therapeutic effects observed in inflammatory diseases (31, 32). The proposed mechanism by which the KD exerts anti-inflammatory effects in COPD is illustrated in Figure 2. While these findings are promising, further research is needed to establish the long-term safety, efficacy, and clinical applicability of KD in COPD management.

This study seeks to explore the perceptions of healthcare professionals in Saudi Arabia regarding the potential therapeutic role of the KD in COPD management. Specifically, the study aims to identify the benefits, challenges, and barriers to adopting KD as part of COPD treatment protocols. Additionally, it examines predictors of KD training uptake and the likelihood of discussing dietary interventions with COPD patients through logistic regression analysis.

## 2 Methods

### 2.1 Ethical consideration

Ethical approval was obtained from an independent research committee at King Faisal University (ID: ETHICS2220).

### 2.2 Study design and participants

This study used an online survey to explore healthcare professionals' perceptions of the KD among patients with COPD. The survey was conducted from June 1, 2024, to September 30, 2024. Healthcare practitioners were recruited using a convenience sampling method. The target population included health professionals likely to be involved in the care of patients with COPD, such as nurses, dietitians, family physicians, respiratory therapists, and general practitioners. Before participating, all potential respondents were informed about the study's objectives. Participation was entirely voluntary, and respondents were assured they could withdraw at any time without consequences. Confidentiality of the collected data was guaranteed, and no personal information was shared. Participants provided consent by responding “yes” to the question, “Do you agree to participate in the study?”. Completing the survey required ~4–5 min.

To maximize participant reach, the researchers collaborated with professional committees, including the Saudi Society for Clinical Nutrition, the Saudi Society for Respiratory Care, and the Saudi Society for Family and Community Medicine. These organizations distributed the questionnaire via email to their members. In addition, the survey link was shared on social media platforms, including X (formerly Twitter), WhatsApp, and Telegram, to enhance participation and diversify the sample.

### 2.3 Questionnaire tool

The questionnaire consisted of four sections with closed-ended questions and optional free-text responses. It was developed by an independent researcher (SA) and reviewed by another researcher (EEA). A pilot study involving 50 healthcare practitioners (family physicians, pulmonologists, general practitioners, nurses, and dietitians) was conducted to validate the tool. Pilot participants were excluded from the final analysis.

1. **Section 1**: collected demographic and professional information, including gender, age, geographic location, specialty, clinical experience (in years), average number of COPD patients seen per month, prior training on the KD, and knowledge of the KD.
2. **Section 2**: contained 10 Likert-scale statements (1 = strongly disagree, 5 = strongly agree) to evaluate participants' opinions on prescribing the KD for COPD patients.
3. **Section 3**: focused on current practices in managing COPD patients, addressing dietary discussions, their frequency, common dietary recommendations, and factors influencing decisions to recommend or not recommend the KD.
4. **Section 4**: included two questions exploring perceived facilitators and barriers to prescribing the KD for COPD patients.

### 2.4 Statistical analysis

Data analysis was performed using JASP version 0.17.2. Descriptive statistical methods, including frequencies and percentages for categorical variables, were used to summarize the survey responses. Additionally, logistic regression analysis was conducted to identify predictors of receiving KD training and discussing dietary interventions with COPD patients. This analysis evaluated relationships between multiple independent variables, such as familiarity with KD, age, and professional background, and the dependent outcomes. GraphPad Prism (GraphPad Software, San Diego, CA, USA) was used to generate graphs.

## 3 Results

### 3.1 Characteristics of the respondents

A total of 1,068 healthcare professionals completed the online survey. The majority of respondents (64%) worked in government hospitals. They were categorized by specialty as follows: dietitians (27%), registered nurses (17%), pulmonologists (16%), family medicine practitioners (14%), general practitioners (16%), and pharmacists (10%). In terms of clinical experience, 37% of respondents reported having 1 to 4 years of experience in COPD care, with most seeing an average of 1 to 3 COPD patients per month. Notably, 68% of participants indicated they had no formal education or training on the KD. Despite this, 76% of respondents reported being somewhat familiar with the KD. A detailed summary of respondent characteristics is provided in Table 1.

**Table 1:** Demographics and professional background of study respondents (*n* = 1,068).

**Characteristics**	**Frequency (%)**
**Gender**	
Male	544 (51)
Female	524 (49)
**Geographical region**	
Central region	168 (16)
Eastern region	186 (17)
North region	256 (24)
South region	215 (20)
Western region	243 (23)
**Profession**	
Dietitian	286 (27)
Family medicine	151 (14)
General practitioner GP	168 (16)
Pharmacist	111 (10)
Pulmonologist/respiratory medicine	171 (16)
Registered nurse	181 (17)
**Primary place of work**	
Governmental hospital	688 (64)
Private hospital	380 (36)
**Years of clinical experience with COPD**	
≤ 1 year	204 (19)
1–4 years	394 (37)
5–9 years	325 (30)
≥ 10 years	145 (14)
**The average number of COPD patients seen per month**	
0	104 (10)
1–3	548 (51)
4–6	281 (26)
7–9	86 (8)
≥ 10	49 (5)
**Received specific formal education or training on the ketogenic**	
**diet**	
No	725 (68)
Yes	343 (32)
**Familiarity with the ketogenic diet**	
Not familiar	55 (5)
Somewhat familiar	807 (76)
Very familiar	206 (19)

COPD, Chronic obstructive pulmonary disease; KD, ketogenic diet.

### 3.2 Healthcare professional' views on the KD in COPD patients

Among the 1,068 respondents, 58% believed that the KD could improve the quality of life in COPD patients. Similarly, 61% agreed that the KD may exert anti-inflammatory effects, potentially benefiting COPD patients with lung inflammation. Moreover, 61% believed that the KD could help alleviate specific COPD symptoms, such as dyspnoea and fatigue.

In addition, 73% agreed that the KD might aid in weight management for COPD patients, which is critical, as obesity can exacerbate symptoms and increase the risk of complications. However, 66% expressed concern that the high-fat content of the KD might lead to increased carbon dioxide production, potentially worsening respiratory symptoms in COPD patients.

Furthermore, 76% agreed with the potential side effects of the KD, such as constipation, dehydration, or electrolyte imbalances, which could exacerbate pre-existing health issues in COPD patients. Meanwhile, 73% believed that restricting carbohydrates, a primary source of essential nutrients and fiber, may increase the risk of micronutrient deficiencies in COPD patients, potentially affecting overall health and immune function.

**Figure 3:**
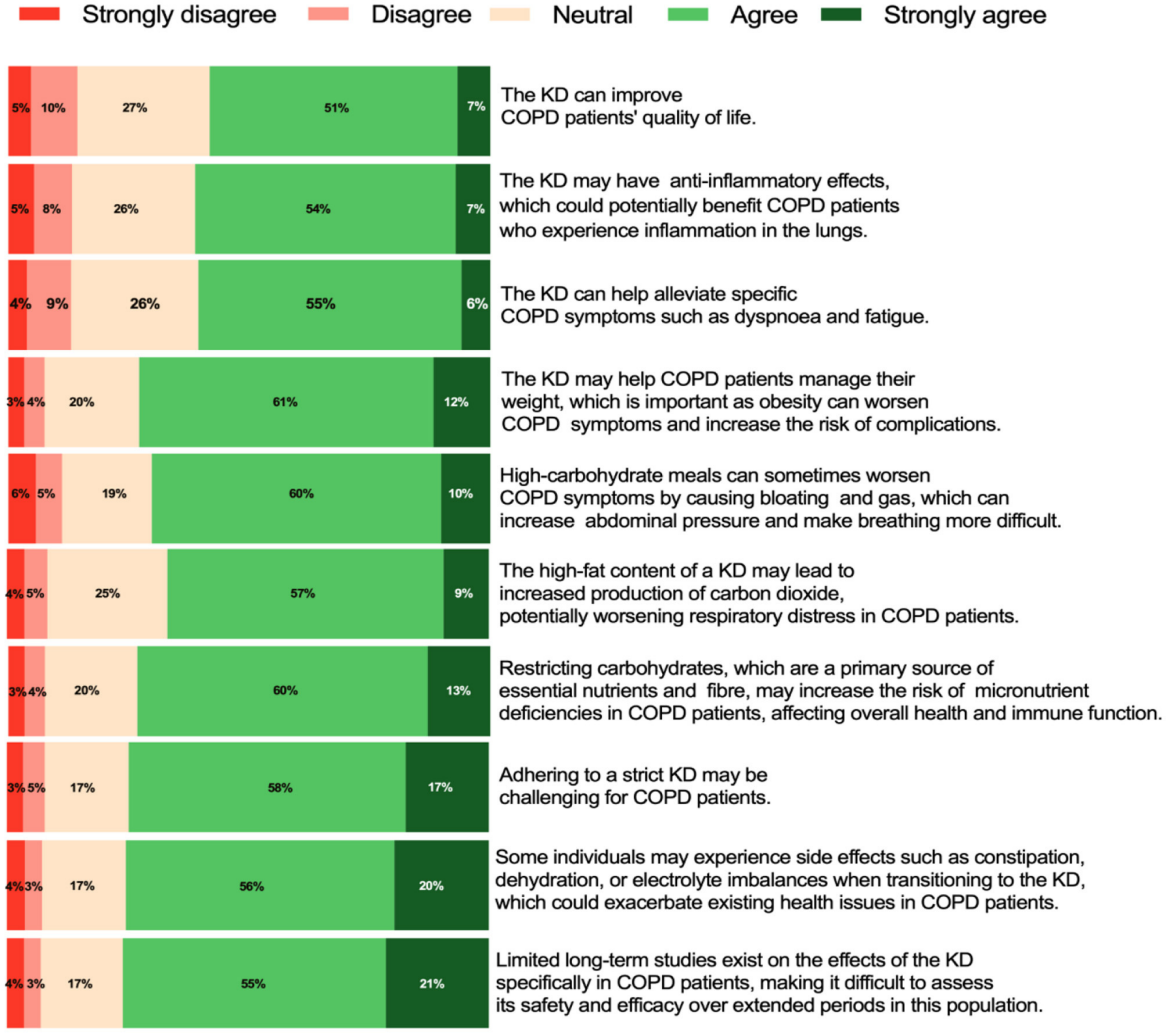
Health professionals' views on the ketogenic diet in patients COPD (*n* = 1,068).

Conversely, 70% agreed that high-carbohydrate meals could worsen COPD symptoms by causing bloating and gas, which may increase abdominal pressure and make breathing more difficult. Additionally, 74% agreed that adhering to the strict requirements of the KD might be challenging for COPD patients. Finally, 76% agreed that the lack of long-term studies on the effects of the KD in COPD patients makes it difficult to assess its safety and efficacy over extended periods for this population. Figure 3 provides a summary of healthcare professionals' views on the KD in COPD patients.

### 3.3 Current practices and attitudes toward recommending the KD to COPD patients

Only 51% of respondents reported discussing dietary interventions with COPD patients, and among these, the KD was recommended by only 14%. Most respondents (67%) prioritized a balanced diet, while smaller proportions advocated for high-protein (11%) or Mediterranean diets (8%). These results suggest that despite growing awareness of the KD, it remains underutilized in clinical practice for COPD patients. This could be due to limited evidence or insufficient professional training. Table 2 provides an overview of dietary practices and attitudes.

**Table 2:** Current practices and attitudes toward recommending the KD to COPD patients (*n* = 1,068).

**Practices**	**Frequency (%)**
**Discusses dietary intervention with COPD patients**	
No	522 (49)
Yes	546 (51)
**Frequency of dietary discussions with COPD patients**	
Never discuss dietary interventions and always transfer patients to a dietitian	320 (30)
Never, dietary interventions are not typically discussed	147 (14)
Occasionally, based on patient interest or need	302 (28)
Rarely, only if specifically requested by the patient	215 (20)
Routinely, during every patient visit	84 (8)
**Primary dietary recommendations for COPD patients**	
Balanced diet	720 (67)
High protein diet	115 (11)
Ketogenic diet	147 (14)
Mediterranean diet	86 (8)

Definitions used in this study: a balanced diet provides moderate proportions of macronutrients based on general guidelines; a high-protein diet includes ≥20% of total energy from protein; the ketogenic diet is characterized by very low carbohydrates ( ≤ 10%) and high fat; the Mediterranean diet emphasizes unsaturated fats, plant-based foods, and moderate protein/carbohydrate intake. COPD, Chronic obstructive pulmonary disease; KD, ketogenic diet.

### 3.4 Barriers to implementing the KD in COPD care

**Figure 4:**
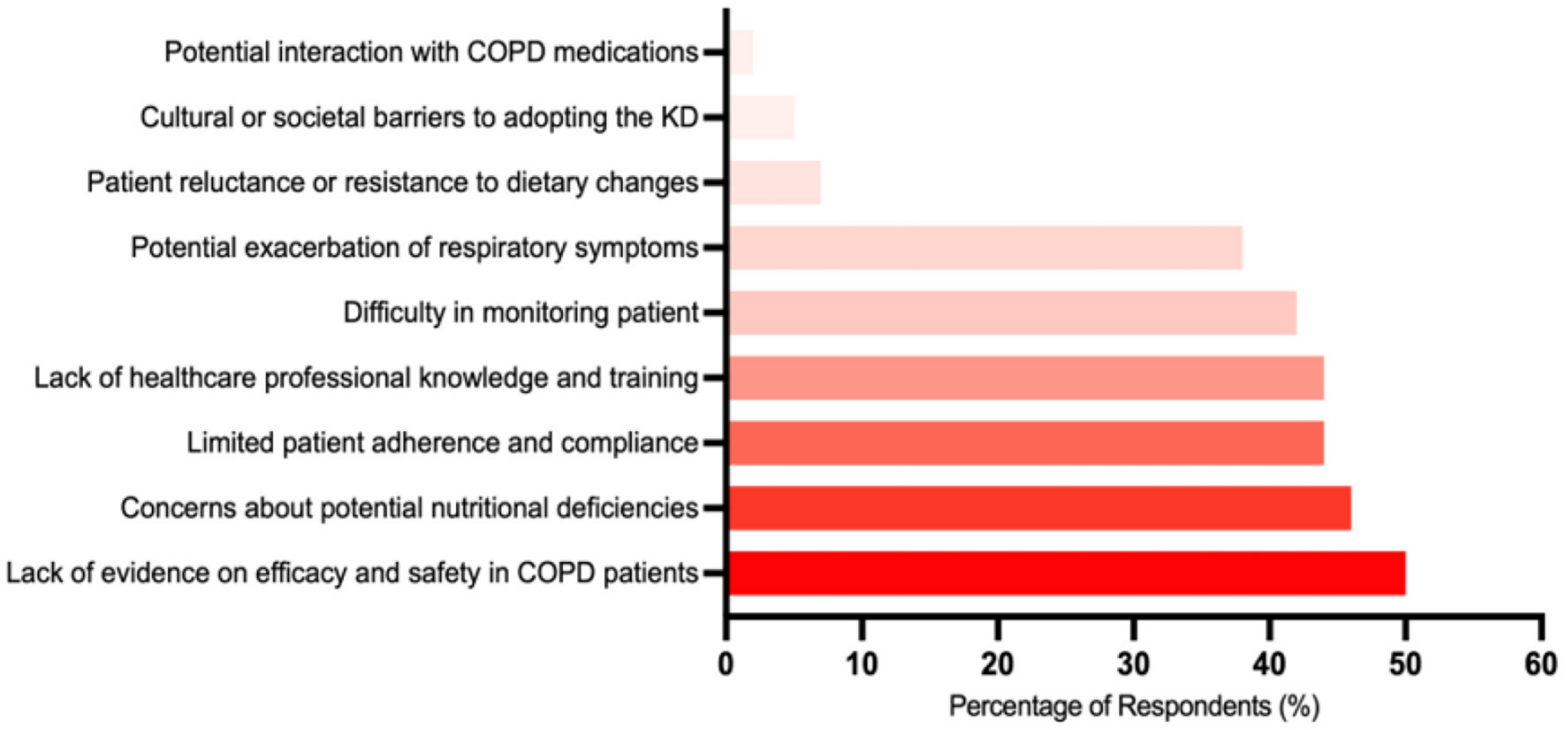
The most common barriers to implementing the ketogenic diet in COPD care (*n* = 1,068).

Respondents were asked to select all applicable barriers to implementing the KD in COPD care from a predefined list. The most commonly selected barrier was the lack of evidence regarding the efficacy and safety of the KD in COPD patients (50%). This was followed by concerns about potential nutritional deficiencies associated with the KD (46%), a lack of healthcare professional knowledge and training (44%), limited patient adherence and compliance (44%), and difficulties in monitoring patient progress and dietary intake (42%). Figure 4 illustrates the distribution of these selected barriers.

### 3.5 Factors that facilitate the integration of the KD into COPD management

**Figure 5:**
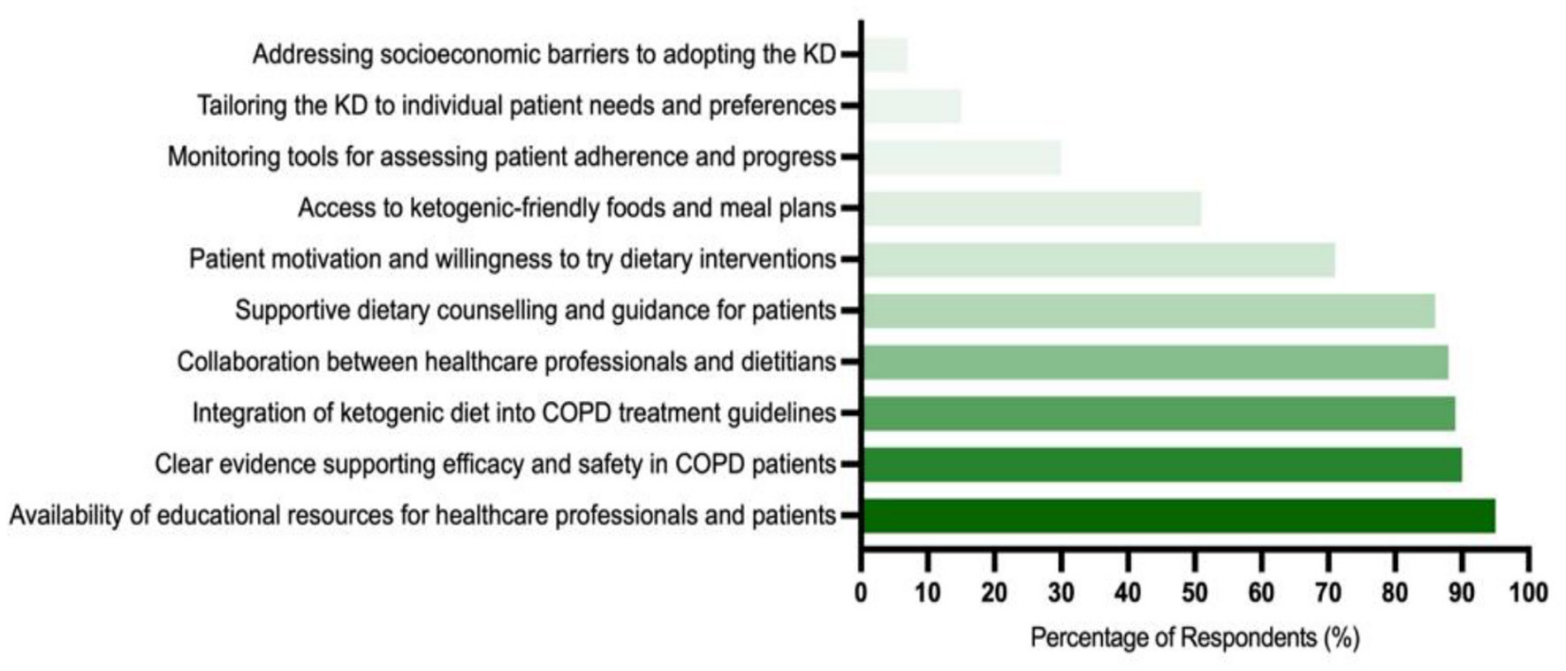
The most common factors that facilitate the integration of the ketogenic diet into COPD management (*n* = 1,068).

The most selected factor was the availability of educational resources for both healthcare professionals and patients (95%). This was followed by clear evidence supporting the efficacy and safety of the KD in COPD patients (90%), the inclusion of the KD in COPD treatment guidelines (89%), collaboration between healthcare professionals and dietitians (88%), and supportive dietary counseling and guidance for patients (86%). Figure 5 provides the percentages of these selected facilitating factors.

### 3.6 Predictors of receiving formal education or training on the KD

As shown in Table 3, logistic regression analysis indicates that familiarity with the KD emerged as a significant predictor. Professionals who reported familiarity with the KD were 21 times more likely to have received formal training (odds ratio = 21.159, *p* < 0.001).

**Table 3:** Logistic regression results for predictors of receiving KD training or education.

					**Wald test**			**95% Confidence interval**	

**Variable**	**Estimate**	**Standard error**	**Odds ratio**	***z***	**Wald statistic**	**df**	***p***	**Lower bound**	**Upper bound**
Professional background (general practitioner GP)	−0.124	0.307	0.883	−0.406	0.165	1	0.685	0.725	0.477
Professional background (pulmonologist/respiratory medicine)	−0.007	0.319	0.993	−0.021	4.621 × 10^−4^	1	0.983	0.633	0.619
Professional background (dietitian)	0.848	0.294	2.335	2.883	8.309	1	0.004	0.271	1.424
Professional background (registered nurse)	0.454	0.305	1.574	1.486	2.209	1	0.137	0.145	1.052
Professional background (family medicine)	0.282	0.331	1.326	0.853	0.728	1	0.393	0.366	0.931
Workplace (private hospital)	−0.035	0.178	0.966	−0.194	0.038	1	0.846	0.384	0.315
Geographical region (North region)	0.658	0.264	1.931	2.491	6.206	1	0.013	0.140	1.176
Geographical region (central region)	0.396	0.274	1.486	1.443	2.083	1	0.149	0.142	0.934
Geographical region (south region)	0.466	0.266	1.593	1.747	3.054	1	0.081	0.057	0.988
Geographical region (western region)	0.035	0.245	1.035	0.141	0.020	1	0.888	0.446	0.515
Gender (female)	−0.260	0.161	0.771	−1.617	2.613	1	0.106	0.575	0.055
Familiarity with KD (somewhat familiar)	3.077	0.217	21.690	14.204	201.752	1	**< 0.001**	2.652	3.501
Familiarity with KD (not familiar)	2.793	0.365	16.327	7.652	58.551	1	**< 0.001**	2.077	3.508
Experience with COPD	0.144	0.095	1.155	1.523	2.319	1	0.128	0.041	0.330

“No” was used as the reference category in the regression model for the variable “Received specific formal education or training on the ketogenic diet.” Bold values indicate statistically significant results at *p* < 0.05.

### 3.7 Predictors of dietary intervention discussions in COPD care

As shown in Table 4, logistic regression analysis showed statistically significant effects of some factors associated with discussing dietary interventions with COPD patients. Nutrition professionals were more likely to discuss dietary interventions than GPs, with an odds ratio (OR) of 1.418 (95% CI: 1.106–1.730, *p* < 0.001). In contrast, respiratory professionals were less likely to discuss, with an OR of 0.618 (95% CI: 0.502–0.731, *p* < 0.001). Knowledge of the KD showed a significant positive effect, with the likelihood of discussion increasing with knowledge, with an OR of 1.402 (95% CI: 1.207–1.627, *p* < 0.001). Similarly, previous experience with COPD patients was associated with an increased likelihood of discussing dietary interventions, with an odds ratio of 1.522 (95% CI: 1.187–1.948, *p* < 0.001).

**Table 4:** Logistic regression results for predictors of dietary intervention discussion in COPD patients.

					**Wald test**			**95% confidence interval**	
**Variable**	**Estimate**	**Standard error**	**Odds ratio**	**z**	**Wald statistic**	**df**	***p***	**Lower bound**	**Upper bound**
Workplace (private hospital)	0.337	0.166	1.401	2.032	4.130	1	0.042	0.012	0.662
Professional background (general practitioner GP)	−0.618	0.266	0.539	−2.320	5.384	1	0.020	−1.140	−0.096
Professional background (pulmonologist/respiratory medicine)	−0.152	0.281	0.859	−0.539	0.291	1	0.590	−0.703	0.400
Professional background (Dietitian)	−2.837	0.290	0.059	−9.787	95.782	1	**< 0.001**	−3.405	−2.269
Professional background (registered nurse)	0.112	0.261	1.118	0.429	0.184	1	0.668	−0.399	0.623
Professional background (family medicine)	0.339	0.291	1.404	1.163	1.353	1	0.245	−0.232	0.910
Geographical region (north region)	0.237	0.239	1.268	0.991	0.983	1	0.322	−0.232	0.706
Geographical region (central region)	0.632	0.253	1.882	2.496	6.232	1	0.013	0.136	1.129
Geographical region (south region)	0.289	0.245	1.335	1.179	1.391	1	0.238	−0.191	0.770
Geographical region (western region)	0.275	0.234	1.317	1.176	1.383	1	0.240	−0.184	0.735
Familiarity with KD (somewhat familiar)	0.777	0.190	2.175	4.100	16.810	1	**< 0.001**	0.406	1.148
Familiarity with KD (not familiar)	0.747	0.338	2.111	2.211	4.889	1	0.027	0.085	1.410
Experience with COPD	0.420	0.086	1.522	4.872	23.738	1	**< 0.001**	0.251	0.589

“No” was used as the reference category in the regression model for the variable “Discusses dietary intervention with COPD patients.” Bold values indicate statistically significant results at *p* < 0.05.

## 4 Discussion

This study aimed to evaluate healthcare professionals' perceptions regarding the KD as a potential therapeutic approach for managing COPD. The findings offer valuable insights into the perceived benefits, concerns, and barriers associated with KD implementation in COPD care. These perspectives highlight both the promise of KD and the challenges that need to be addressed for effective integration into clinical practice.

A significant portion of respondents (58%) acknowledged the potential of KD to enhance COPD patients' quality of life, primarily due to its anti-inflammatory effects. This perception aligns with emerging evidence suggesting that beta-hydroxybutyrate (BHB), a key ketone body produced during ketosis, can inhibit the NLRP3 inflammasome—a critical component of chronic inflammation in COPD pathophysiology (33, 34). Additionally, KD has been shown to suppress the NF-κB signaling pathway, reducing pro-inflammatory cytokines such as IL-1β, TNF-α, and IL-6 (34, 35). These mechanisms provide a plausible rationale for the anti-inflammatory benefits of KD in COPD management.

Further clinical evidence supports KD's role in improving mitochondrial efficiency, reducing oxidative stress, and enhancing respiratory function (33, 35). These combined effects suggest that KD could address multiple aspects of COPD pathology, including inflammation, energy metabolism, and respiratory efficiency. However, while these findings are promising, they underscore the necessity for well-designed, long-term clinical trials to confirm the efficacy and safety of KD in COPD management and determine its impact on patient-reported outcomes.

Despite the perceived benefits, 66% of respondents expressed concerns about KD's high-fat content and its potential to increase carbon dioxide (CO_2_) production, which could worsen respiratory distress. While such concerns are theoretically valid, evidence indicates that a low-carbohydrate, high-fat diet may reduce CO_2_ production due to a lower respiratory quotient (RQ) associated with fat metabolism (36–38). This discrepancy highlights a critical need for more robust research and clear guidelines to reconcile differing viewpoints and provide definitive recommendations for clinical practice.

Several barriers to adopting KD in COPD care were identified. The most frequently cited challenge was patient adherence (74% of respondents). KD's restrictive nature, coupled with side effects such as fatigue, gastrointestinal discomfort, and social limitations, presents significant hurdles to long-term compliance (21, 39). These challenges necessitate the development of personalized dietary plans and ongoing support mechanisms to improve adherence and mitigate side effects.

Another notable barrier was the lack of professional training in KD implementation (44% of respondents). This knowledge gap limits healthcare providers' ability to educate and guide patients effectively. Furthermore, 76% of respondents highlighted the absence of long-term studies on KD's safety and efficacy in COPD management, emphasizing the urgent need for further clinical research to build a comprehensive evidence base.

To address these barriers, several facilitators must be prioritized. A majority (95%) of respondents stressed the importance of comprehensive educational programs for both healthcare professionals and patients. These programs should focus on the practical aspects of KD implementation, the potential benefits, and strategies to manage side effects. Effective education can empower healthcare providers to deliver evidence-based dietary advice and support patients through the challenges of adhering to KD.

Additionally, robust scientific evidence was identified as critical by 90% of respondents. Collaborative efforts among clinicians, researchers, and policymakers are essential to generate high-quality evidence and develop standardized clinical guidelines for KD use in COPD management. This will help bridge the gap between theoretical knowledge and clinical application, fostering greater confidence in KD as a therapeutic option.

Logistic regression analysis identified familiarity with the KD as a key factor in determining interest in training, with results showing that participants with partial knowledge of the diet were 21 times more likely to receive training than those without (*p* < 0.001). This strong association suggests that awareness of the KD should be raised among healthcare professionals to enhance training opportunities and increase implementation of this strategy in clinical care. Previous studies confirm that targeted nutrition education enhances healthcare professionals' confidence and competence in implementing nutritional interventions (40). Therefore, incorporating experiential learning and interactive training modules into professional education could facilitate the adoption of the KD in clinical practice.

Furthermore, logistic regression analysis highlighted the role of healthcare professionals' backgrounds in determining the likelihood of discussing nutritional interventions. Dietitians were significantly more likely to engage in discussions about the KD with COPD patients compared to general practitioners (*p* < 0.001). Conversely, respiratory specialists were less likely to discuss nutritional interventions, which may reflect their greater focus on lung-specific treatments rather than holistic approaches, including nutrition. This disparity underscores the need for multidisciplinary education and training programs to bridge the knowledge gap between respiratory care and medical nutrition, and thus promote collaborative approaches to COPD management.

## 5 Strengths and limitations

This study presents several strengths. Firstly, it offers novel insights into healthcare professionals' perceptions of the KD as a therapeutic strategy for COPD management, addressing a critical gap in the literature—particularly within the Saudi Arabian healthcare context. Secondly, the application of logistic regression analysis enabled a robust evaluation of associations between participants' familiarity, professional training, and demographic characteristics, thereby enhancing analytical rigor. Thirdly, the relatively large sample size (*n* = 1,068) strengthens the reliability and validity of the findings, offering a representative view of prevailing attitudes toward KD. Finally, the inclusion of a broad range of healthcare professionals—such as general practitioners, dietitians, and nurses—provides a multidimensional perspective on the perceived barriers and facilitators to implementing KD in clinical settings.

Nonetheless, this study has limitations. The cross-sectional design limits causal inference regarding KD's impact on COPD outcomes. Longitudinal studies and randomized controlled trials are required to establish long-term safety and efficacy. Additionally, the use of self-reported data introduces potential biases, including recall, response, and social desirability bias, which may affect data accuracy. The sample was confined to healthcare professionals in Saudi Arabia, which may limit the generalizability of results to broader international contexts. Differences in healthcare infrastructure, nutritional practices, and cultural perceptions may affect the external applicability of the findings. Future research in diverse populations is essential to validate and extend these insights. Moreover, the study focused on perceptions rather than clinical outcomes, precluding definitive conclusions about KD's effectiveness in COPD symptom management. Finally, insufficient training and knowledge among respondents may have influenced their responses. These limitations underscore the need for comprehensive educational programs, rigorous clinical trials, and diverse participant cohorts in future investigations.

## 6 Conclusion

This study highlights the therapeutic potential of the ketogenic diet in the context of COPD management, particularly with regard to its anti-inflammatory and symptom-modifying effects. However, several practical challenges remain, including patient adherence, potential side effects, and gaps in professional training. It is also essential to distinguish between healthcare professionals who might suggest the ketogenic diet as a nutritional approach and those—such as registered dietitians—qualified to prescribe and supervise individualized diets.

These findings have practical implications for clinical practice, forming the basis for developing context-sensitive guidelines and multidisciplinary educational initiatives. Strengthening evidence-based knowledge and improving competency among healthcare providers could support the safe and effective integration of the ketogenic diet into the care of patients with COPD. Furthermore, international studies are needed to assess the consistency of the patterns identified in this study across different health systems and cultural settings. As nutritional strategies grow in popularity in chronic disease management, attention to healthcare provider readiness and system-wide implementation barriers will be critical to bridging the gap between evidence and practice.

## Data Availability

The original contributions presented in the study are included in the article/supplementary material, further inquiries can be directed to the corresponding author.
